# Efficacy of Supportive Therapy of Allergic Rhinitis by Stinging Nettle *(Urtica dioica)* root extract: a Randomized, Double-Blind, Placebo- Controlled, Clinical Trial

**Published:** 2017

**Authors:** Mehdi Bakhshaee, Amir Hooshang Mohammad pour, Majid Esmaeili, Farahzad Jabbari Azad, Ghazal Alipour Talesh, Maryam Salehi, Morteza Noorollahian Mohajer

**Affiliations:** a *Ear, Nose and Throat * *Research * *Center, Imam Reza * *Hospital, * *Faculty of * *Medicine, * *Mashhad University of Medical Sciences, Mashhad, Iran. *; b *Pharmaceutical Research Center, Mashhad University of Medical Sciences, Mashhad, Iran.*; c *Department of Clinical Pharmacy, School of Pharmacy, Mashhad University of Medical Sciences, Mashhad, Iran. *; d *Allergy Research Center, Ghaem Hospital Faculty of Medicine, Mashhad University of Medical Sciences, Mashhad, Iran.*; e *Nanotechnology Research Centre, School Pharmacy,Mashhad University of Medical Sciences, Mashhad, Iran.*; f *Community Medicine Department, Faculty of Medicine Mashhad University of Medical Sciences, Mashhad, Iran.*

**Keywords:** allergic rhinitis, Nettle, *Urtica Dioica*, cytokine

## Abstract

The aim of this study was to survey the exact benefit of this herb in the management of clinical and laboratory signs and symptoms of allergic rhinitis. In a randomized double blind clinical trial, 74 patients with the signs and symptoms of allergic rhinitis and a positive skin prick test were selected and randomly divided into 2 groups who were *taken Urtica dioica* 150-mg, Urtidin^® ^F.C Tablet) or placebo for one month. Their signs and symptoms, eosinophil percentage on nasal smear, serum IgE, and interleukin IL-4, IL-5, interferon- γ) levels were recorded.

Forty patients completed the trial. Based on the Sino- Nasal Outcome Test 22 SNOT-22), a significant improvement in clinical symptom severity was observed in both groups *P *< .001). Furthermore, a statistically significant reduction in mean nasal smear eosinophil count was observed after treatment with Nettle *P *< .01). However, the mean IgE and IL4 and IL5 levels in the study group before and after treatment with Nettle saw no significant changes *P *> .1).

Intergroup pre- and post-treatment laboratory findings suggested that there was a significant difference in post-treatment changes of mean IFN γ levels between the study and placebo group P = 0.017). Although the current study showed certain positive effects of Nettle in the management of allergic rhinitis on controlling the symptoms based on the SNOT-22, similar effects were demonstrated by placebo as well. We believe that our limitations underscore the need for larger, longer term studies of Nettle for the treatment of allergic rhinitis.

## Introduction

Allergic rhinitis has the most common allergic disorder in various regions, affecting 20 percent or more of the general population (1-3). This figure continues to risen though (4). Persistent allergic rhinitis was an IgE-mediated inflammatory disorder of the nasal mucosa characterized by continuous symptoms presented for more than 4 days a week consecutive weeks) nasal congestion, rhinorrhea, sneezing, itching, purities of the conjunctiva, nasal mucosa and oropharynx, allergic shiners, lacrimation, along with ocular symptoms and fatigue (5). The condition could be caused by environmental agents such as dust mites, mold, pollen, fungus spores, cockroaches, grass, animal dander, feathers, and also food sensitivities, structural abnormalities, metabolic conditions, or drugs (2, 6). Allergen avoidance has the essential component of allergy management but was not always practical for all patients (5). There was a wide range of over-the-counter OTC) medications on the market for potential allergy management. However, many of these were accompanied with adverse side-effects like sedation, headache, dry mouth, drowsiness, impaired learning/memory, and cardiac arrhythmias, projecting the need for newer therapeutic strategies that could decrease the morbidity associated with Allergic Rhinitis and already in-use drug regimens. On the other hand, various studies have demonstrated that rhinitis patients were tormented by repeated nose blowing, had a disrupted sleep pattern, were fatigued, suffered from a significant decline in concentration, verbal learning, decision-making speed, and psychomotor speed which in turn might lead to considerable reduction in productivity level at work, frequent absenteeism from work and school and also a significant decline in general quality of life (7-11). As the financial costs and the negative impact of allergic rhinitis on life quality were of high importance and it was documented as a major risk factor for developing future asthma (12), and effective treatment would be extremely valuable.

In recent years, significant changes have occurred in the strategies of allergic rhinitis treatment (13). On the other hand, there has been a growing trend towards using herbal therapy for both medical and economic reasons (14). It has been reported that herbal therapy was frequently used for the treatment of allergic diseases in various parts of the world (15-17). Based on these facts, there was a need among general physicians, otolaryngologists and immunologists for more knowledge about herbal therapies (18) and for pharmaceutical scientists to further investigate and document the actual efficacy of such treatments.

Nettle *Urtica dioica *L., Family: Urticaceae) was a well-known medicinal plant that has long been used worldwide in complementary and alternative medicine (19). It was native to Eurasia and was widely distributed throughout the temperate regions of the world, including the U.S (20). Nettle which was a perennial, temperate dioecious plant, prefers wet, nutrient rich soil, lighted places, hot and mild climate and tends to grow in large patches (21). It has 2–4 cm long, oval, core shape, fleshy, drooping, opposite, sharply toothed leaves. The leaves and stems were covered with persistent stinging nettle. It produces inconspicuous small green-white flowers from May to September. The fruits of nettle were arid and single germ (21, 22). In recent years, several studies have been conducted to examine and confirm the medicinal properties of Nettle and investigate the underlying biochemical mechanisms of such activities. Nevertheless, confirmatory clinical trials in humans were yet needed. Nettle extensive use in medicine and also the entry of its products such as oral capsules and topical solutions either alone or in combination with other herbs) to the pharmaceutical industry (23, 24), provoke the need for a precise knowledge of the herb’s pharmacological properties. An overview on the different uses of this herb and the most relevant active ingredients was provided in Table 1.

To our interest were the reported anti-oxidant and anti-inflammatory properties of Nettle which are investigated and proven in several studies. The role of oxidants and oxidative stresses in the pathophysiology of allergic rhinitis has been confirmed in several studies (25, 26). Mittman *et al*., (27) reported that while the freeze-dried extract of nettle leaves reduced allergy symptoms based on Global assessment at the end of the double blind clinical trial, only a small difference was observed between the herb and placebo on the daily response diaries. Based on such controversial evidences, we faced lack of sufficient, recent proof to support or refute the use of *Urtica Dioica* in the treatment of allergic rhinitis. Herein, we aimed to survey the exact benefit of 

this herb in the management of clinical and laboratory signs and symptoms of allergic rhinitis. 

**Table 1:** An overview of reported pharmacological effects of *Urtica dioica*

**Effect**	**Induced by**	**Part of plant**	**References**
Allergic Rhinitis		Leaves	(21)
Antiproliferative effects on human prostate cancer cells: treatment of benign prostatic hyperplasia (BPH)	root lignans, such as (-)-3,4-divanillyltetrahydrofuran	Root	(34-36)
Anti-inflammatory	Polysaccharides and caffeic malic acid (CMA)/ cyclooxygenase and lipoxygenase inhibition	All Parts	(37, 38)
Supportive therapy in Cardiovascular diseases	Inhibition of thrombin-induced platelet aggregation and improving lipid profiles (decreasing total and LDL cholesterol, plasma Apo B, and LDL/HDL ratio) Flavonoids	Leaves	(39, 40)
Supportive therapy in lower urinary tract infections and urinary gravel		Leaves	(16)
Arthritis, lumbalgia, sciatica, chronic tendonitis, sprains and osteoarthritis.		Leaves	(41, 42)
Antioxidant, antimicotic and antibacterial activity	phenolic compounds	Leaves	(43)
Immunostimulatory activity on neutrophils, Supportive therapy of neutrophil function deficiency and chronic granulomatous diseases	quercetin and isorhamnetin glycosides	Aerial Parts	(44)

## Patients and Methods

**Figure 1:**
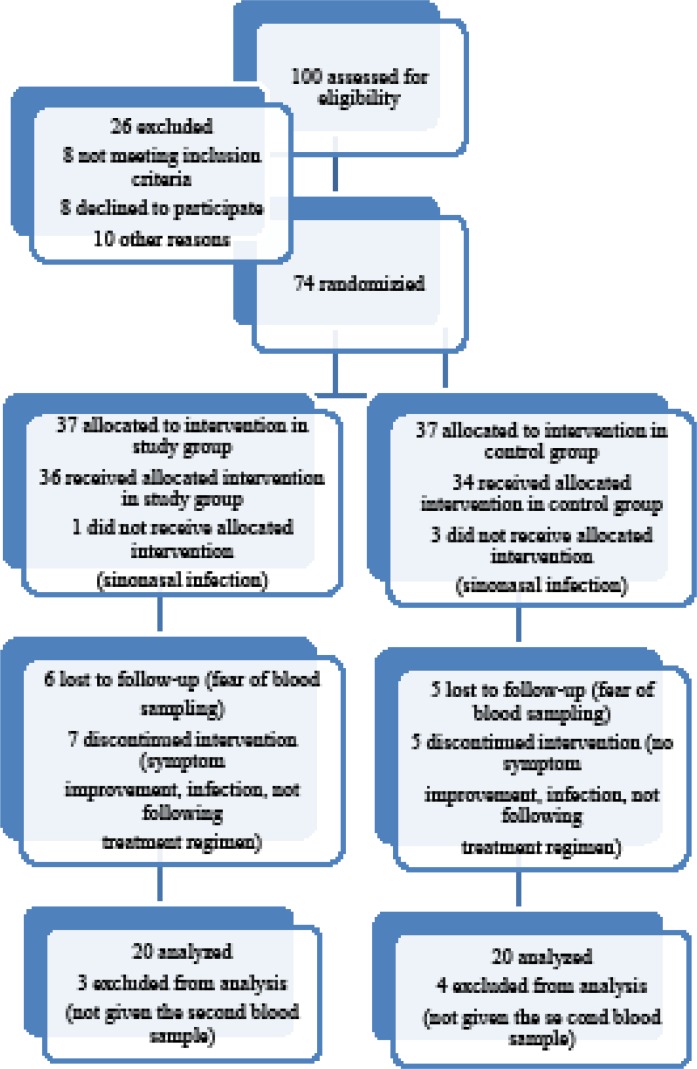
Flow diagram of the patients in the study and control groups

This study was randomized, double blind clinical trial which evaluated the additive effect of Nettle on reducing the signs and symptoms of allergic rhinitis. In this study 100 patients with allergic rhinitis who visited the Allergy and the Ear, Nose, and Throat ENT) clinic of Qaem educational hospital, Mashad, Iran, from June 2013 to April 2014 were assessed for eligibility. This clinical trial was registered in www.irct.ir, with IRCT 2013102715177N1 number and was approved by the ethics committees of Mashhad University of Medical Sciences MUMS). For all patients consent information filled to the participation in the record according to the requirements of the ethics committee within each hospital . Of the 74 patients who fully met the inclusion criteria, 40 patients completed the trial and performed laboratory tests at both time points Figure 1). Because the present work was a pilot study at the time of initiation, we chose the sample size based on the number of referral cases to our clinics and the available material for performing such a study. The inclusion criteria for this study was having the clinical signs/symptoms of allergic rhinitis and a positive allergy skin test for at least one of the tested allergens based on the studies that have investigated the prevalence of allergy and common allergens in this region, a total of 18 common regional allergens were tested and reported in 4 categories: tree, mixed grass, *Salsola *(weed), and *Alternaria *(mold)). Persistent allergic rhinitis was defined as related symptoms present for much than 4 days a week and consecutive weeks, with the realization that patients usually suffer almost every day. For all these patients, oral antihistamines H1 type) and also inhaled corticosteroids had been previously administered according to the standard treatment protocol of allergic rhinitis, but patients had experienced no or very little clinical response. By doing a simple randomization method, the cases were divided into 2 groups 40 cases each); both groups also received the routine daily treatment for allergic rhinitis: Loratadine10 mg Shahid Ghazi Pharmaceutical Co., Tabriz, Iran) daily and nasal Saline rinses Tehran Chemie Pharmaceutical Co., Tehran, Iran) 3 times a day. Along with this therapy, a 1-month treatment course of Nettle was administered as 150-mg F.C. tablets Urtidin^®^, Barij Essence Pharmaceutical Co., Kashan, Iran) prescribed 4 times daily for the study group. The control group received placebo for the same duration. The placebo was prepared from excipients that were used as conservatives or carriers besides the main therapeutic components and matched for size, shape, and volume of contents and manufactured by the same company. This experiment was approved by the Ethics Committee of Mashhad University of Medical Sciences; all patients were fully informed about the study protocol, and a signed informed consent was obtained from each of them. Patients with any other etiology for rhinitis, or with other underlying systemic diseases, and those taking any kind of herbal drugs with antioxidant effects were excluded from the study. The clinical symptom severity of the patients was evaluated by the standard Sino-Nasal Outcome Test 20 SNOT-22) questionnaire. A skin test for all the common regional aeroallergens— tree, mixed grass, *Salsola *weed), and *Alternaria* mold)—was also performed for each case to confirm the allergic base of the disease. Laboratory tests were performed as previously described (28). The percentage of eosinophils on the nasal smear was then quantified by the use of a high-power field microscope HPF 4); a 5-mL blood sample was collected from each patient at baseline and after 4 weeks end of study period). Plasma was frozen and then stored for analysis at the end of the study. Total blood IgE level was measured through the use of enzyme-linked immunosorbent assay ELISA) kits manufactured by Pishtaz Teb Diagnostics, Tehran, Iran) both at baseline and at the end of the trial. 

Besides, blood samples were obtained to investigate the concentration of interleukin IL)–4 as the major Th2 inducing cytokine), IL-5 eosinophils accumulation inciter), and interferon IFN)–γ, as the major Th1 inducing cytokine): at each time point, a 2-cc blood sample was sent to Bu-Ali immunologic research center Mashhad, Iran), where the lymphocytes were initially separated and cultured for 48h under polyclonal stimulation by implementing the Phycol method Difco, Bacto Laboratories Pty Ltd, Liverpool, England). Once being stimulated with the mitogen agent of Phyto Mito Antigen PMA, which increases the concentration of cytokines and facilitates their measurement), the supernatant was collected and stored at –80°C pending further analysis. At the end of the trial, cell supernatants were analyzed by ELISA assay to measure the cytokine concentrations as directed by the supplier Sanquin Blood Supply Foundation, Amsterdam, the Netherlands). Cytokine data are expressed as the difference between the spontaneous culture and the control pg/mL). After taking the first blood sample by a single physician, all patients were randomly and in a double-blind manner divided into the study and control groups. A simple randomization method was applied where the consecutive patients were divided into the study or control group intermittently. Group selection was by chance coin flip). At the end of the trial, clinical and laboratory signs and symptoms were once again recorded for each patient. Data were then analyzed by applying the SPSS software version 19; SPSS, Inc, an IBM Company, Chicago, Illinois). The pre- and post-treatment changes in each group and between the 2 groups were compared using the paired samples *t *test and independent samples *t *test, respectively.

## Results

We evaluated the clinical and laboratory findings of 40 allergic rhinitis patients divided into 2 groups, both before and after treatment with Nettle. There was not significant difference in terms of age and sex between the 2 groups; while there were 6 30%) men and 14 70%) women in the study group, there were 7 35%) men and 13 65%) women participants in the control group. Mean ±SD) age of the participants in study and control groups was 23.98 ±10.72) years and 28.40 ±10.46) years, respectively.

The prevalence of clinical symptoms among all the studied cases is shown in Table 2. The most frequent symptoms were sneezing and nasal blockage with the frequency rate of 100 and 97.5 percent respectively.

**Table 2:** Prevalence of Dominant Allergic Rhinitis Symptoms in the Studied Cases (SNOT-22

**Symptoms**	**Number **	**Frequency, %**
Sneezing	40	100
Nasal Blockage	39	97.5
Runny nose	38	95
Need to blow nose	38	95
Post-nasal discharge	38	95
Thick Nasal Discharge	35	87.5
Fatigue	74	85
Reduced Concentration	34	85
Reduced Productivity	33	82.5
Lack of a good night’s sleep	31	77.5
Wake up tired	31	77.5
Difficulty falling asleep	29	72.5
Decreased sense of Smell/Taste	29	72.5
Ear fullness	27	67.5
Sad	27	67.5
Wake up at night	25	62.5
Frustrated/restless/irritable	25	62.5
Cough	24	60
Embarrassed	21	52.5
Dizziness	18	45
Facial Pain/Pressure	18	45
Ear Pain	11	27.5

The paired samples *t *test and Wilcoxon signed rank test demonstrated that there was a significant decrease in symptom severity based on SNOT-22) following treatment in the study group *P *< .001). Furthermore, a statistically significant reduction in mean nasal smear eosinophil count was observed after treatment with Nettle (*P *< .01). However, the mean IgE and IL4 and IL5 levels in the study group before and after treatment with Nettle saw no significant changes( *P *>0.1). Mean IFN γ levels experienced a non-significant rise in the study group (*P *= .068). 

Mean clinical symptom severity based on SNOT-22 before and after treatment showed a statistically significant decrease after treatment in the control group *P *< .001). Pre and post treatment mean nasal smear eosinophil count, serum IgE, IL4, and IL5 levels in the control group also demonstrated no significant difference after treatment with placebo *P *> .1). IFN γ levels significantly reduced the following placebo treatment (*P *= .023). The results of Comparing Pre- and Posttreatment Clinical and Laboratory Findings were shown in Table 3.

**Table 3:** Comparing Pre- and Posttreatment Clinical and Laboratory Findings

Study Group				**Placebo Group**		
	**Pre-treatment** **(Mean±SD)**	**Post-treatment** **(Mean±SD)**	**P value**	**Pre-treatment** **(Mean±SD)**	**Post-treatment** **(Mean±SD)**	**P value**
**symptom severity (SNOT-22)**	44.35 ± 14.78	23.10 ± 16.85	<.001	43.5 ± 17.46	27.55 ± 14.55	<.001
**nasal smear eosinophil, %**	19.5 ± 27.70	10.40 ± 19.80	.01	12.50 ± 20.04	10.95 ± 25.00	.405
**serum IgE level**	193.50± 160.73	183.65± 166.83	.37	187.35± 171.31	206.00± 153.85	.911
**interferon-γ level**	0.67 ± 1.23	1.42 ± 2.36	.068	0.82 ± 1.50	0.27 ± 0.43	.023
**interleukin-4 level**	55.77± 156.55	71.29± 239.03	.84	24.54 ± 14.05	17.74 ± 16.10	.089
**interleukin-5 level**	1.05 ± 4.69	1.90 ± 8.49	.317	1.00 ± 4.47	1.45 ± 6.48	.317

Abbreviation: SNOT-22, Sino-Nasal Outcome Test 22. Values presented as mean ± SD.

The difference in pre- and post-treatment mean IFN γ level with the 95% confidence interval was -0.75 ± 1.96 and -0.55 ± 1.27μg/mL intervention and control groups, respectively Table. 4.). Therefore, intergroup Pre- and Post-treatment laboratory findings suggested that there was a significant difference in post treatment changes of mean IFN γ levels between the 2 groups P = 0.017). Demonstrates there was not significant difference in pre- and post-treatment changes of mean clinical symptom severity, nasal smear eosinophil count, serum IgE, IL-4 and IL-5 levels between the 2 groups. *P *= .25, *P *= .142, *P *= .494, and *P *= .259 and p= .680 respectively). The results of Comparing Intergroup Pre- and Posttreatment Clinical and Laboratory Findings were shown in table 4.

**Table 4:** Comparing Intergroup Pre- and Posttreatment Clinical and Laboratory Findings

	**Study Group**	**Control Group**	**P Value**
**Difference in pre- and posttreatment symptom severity (SNOT-22)**	21.25± 16.22	15.95 ± 12.21	0.25
**Difference in pre- and posttreatment nasal smear eosinophil**	9.10 ±15.68	1.55 ± 16.11	0.142
**Difference in pre- and posttreatment serum IgE level**	9.85± 165.55	(-18.65)± 81.42	0.494
**Difference in pre- and posttreatment interferon-** ** γ** ** level**	(-0.75) ± 1.96	0.55± 1.27	0.017
**Difference in pre- and posttreatment interleukin-4 level**	(-15.51) ± 85.08	6.80 ± 18.79	0.259
**Difference in pre- and posttreatment interleukin-5 level**	(-0.85) ±3.80	(-0.45) ± 2.01	0.680

Abbreviation: SNOT-22, Sino-Nasal Outcome Test 22. Values presented as mean ± SD.

## Discussion

Ayers *et al* (29) have reported that adenine, nicotinamide, synephrine and osthole, found in *Urtica dioica *has anti-inflammatory and anti-allergenic properties. All these compounds were found previously to have significant anti-inflammatory effects. Interestingly, Synephrine which is an alkaloid, has been long used as a nasal decongestant (30) and is used in traditional Chinese medicine for treatment of seasonal allergy and other inflammatory disorders (31). More recently, *Urtica dioica* extract was shown to have inserted *in-vitro *inhibition of several key inflammatory events that cause allergic rhinitis symptoms. These include 1. the antagonist and negative agonist activity against the Histamine-1 H1) receptor which blocks histamine production and release, 2. the inhibition of mast cell tryptase hindering mast cell degranulation and consequent release of a host of pro-inflammatory cytokines and chemokines that lead to the appearance of allergy symptoms, 3. Inhibition of Cyclooxygenase-1 COX-1), Cyclooxygenase-2 COX-2) both key enzymes involved in the induction of many inflammation events associated with allergic rhinitis) and therefore prevention of prostaglandin formation, and 4. Hematopoietic Prostaglandin D2 synthase HPGDS) inhibition, that specifically deters Prostaglandin D2 production, a primary pro-inflammatory mediator in allergic rhinitis (32). However, no recent study has yet been performed on the impact of Nettle with proven antioxidant and anti-inflammatory effects) in the treatment of allergic rhinitis. It should be mentioned that in numerous experiments, other herbal products with established antioxidant and anti-inflammatory effects have been studies in this disease, and a satisfactory result mainly in relieving major clinical symptoms and enhancing the quality of life of such patients has been achieved (28, 33). These motivated us to perform a randomized, double blind clinical trial to investigate the efficacy of supportive therapy of allergic Rhinitis by Urtidin^® ^Tablet.

We found out that the dominant symptoms of allergic rhinitis recorded in our patients were similar to those of previous studies. This mainly included sneezing, nasal congestion and clear, watery rhinorrhea. Sleep pattern disorder was also widely seen in the patients. The severity of clinical symptoms based on the patients’ sex showed there was not significant difference between the 2 sexes. This factor has not been separately included in previous studies. 

Here, apart from assessing the conventional clinical symptoms for both diagnosis and evaluation of the degree of recovery, we evaluated laboratory signs related to allergic rhinitis, for the first time. Elicitation of a Th2 response and the decrease in Th1 response are the typical features of inflammatory processes like allergic diseases (34). The secretion of cytokines by Th2 cells leads to the production of specific IgE antibodies by B lymphocytes. IL4 the major Th2 inducing cytokine) which is a necessary signal to B lymphocytes, induces the synthesis of IgE antibodies by B cells. In addition, IL5 induces the accumulation of eosinophils in tissue which is the hallmark but not the only cause) of allergic inflammation and reported to be the major effector cell involved in chronic or perennial rhinitis.

In the current study, Nettle co-administrated with other routine treatments of allergic rhinitis for 1 month, lead to a significant decrease in the severity of clinical symptoms based on the SNOT-22). Furthermore, nasal smear eosinophil count significantly dropped after treatment with Nettle. Saxena *et al* also reported a significant decrease in the total eosinophil count after treatment with an herbal mixture of Aller-7. 

We observed improvement in clinical symptoms in the control group as well. However, in this group, IFN γ as a Th1 cytokine significantly declined meaning that it could lead to the progress of allergic rhinitis after a while. In other words, the improvement in clinical symptoms in the group which received placebo could be temporary as the decline in Th1 response can further enhance the allergic symptoms. Previously, some studies have demonstrated the role of psychological factors in alleviation or worsening of allergic conditions. Also, based on the predictable, short-term positive psychosomatic effect of placebo in any disease and according to the fact that due to our limitations we could not follow the patients any longer, we cannot report on the effect of adding placebo to the routine treatment regimens of allergic rhinitis. This was not the prior aim of the study. 

As mentioned earlier, serum IgE and cytokine levels have not been measured in any previous study that has administered antioxidant compounds for the treatment of allergic rhinitis. In the current study, their post treatment level shows that there was no significant difference in either group, except for IFN γ). This, however, could not refute the efficacy of treatment. It was already demonstrated that the total serum IgE has low sensitivity 43.9%) as well as limited clinical value when evaluating a patient for allergic rhinitis. Because of limited facilities, we evaluated the immunologic condition of the patients by studying serum IgE and cytokine levels, whereas measuring such factors in the nasal discharge –although yet there was not supported by all clinicians, has a much higher specificity and accuracy in evaluating the severity of allergic conditions limited to the upper airway system. This is due to the fact that other simultaneous systemic inflammatory disorders can also affect serum IgE and cytokine levels. Furthermore, the difference in response rates could be due to remarkable differences in oxidative stress laboratory tests, which were not examined in this study because of our limitations but we recommend their investigation for future researches in this field.

With respect to safety, our results were consistent with past researches in reporting preliminary evidence of no serious, deleterious adverse effects with the systemic administration of Nettle. 

Although this experiment had an arguable outcome, several points should be kept in mind for future similar trials. Most importantly, we could not use Nettle solely for controlling the patients’ symptoms; this was due to ethical considerations and the patients’ probable dissatisfaction with being treated by a single experimental drug. Thus, Nettle was applied along with other routine treatments. Second, as our study was only 1 month in duration, it presented no direct evidence of any potential much more positive effects associated with long-term usage. Third, our trial encompassed only 40 subjects at last which resulted in insufficient statistical power to prove the insignificant changes. Fourth, the outcome of our study applies only to the circumstances of such a study and does not reflect any information about what would the results be if, for instance, patients took other Nettle dosage forms with different pharmacokinetic profiles or were on higher doses. Finally, there are concerns over the ingredients of the pharmaceutical Nettle tablets. We conducted our research based on the assumption that the manufacturer's statements about active ingredients were accurate. However, in future research, it would be logical to consider testing for this prior to administration.

## Conclusion

current study shows certain positive effects of Nettle in the management of allergic rhinitis on controlling the symptoms based on the SNOT-22 and also similar effects was demonstrated by placebo. Hence, the exact efficacy of *Urtica dioica* in this respect could not be determined in this study. We believe that our limitations underscore the need for larger, longer term studies of different pharmaceutical dosage forms of Nettle for the treatment of allergic rhinitis.
